# Theory of mind deficits in Korsakoff's syndrome and alcohol use disorder: Similar deficits but different underlying cognitive processes

**DOI:** 10.1111/acer.70135

**Published:** 2025-08-22

**Authors:** Alice Laniepce, Pierre Maurage, Ludivine Ritz, Céline Boudehent, Nicolas Cabé, Shailendra Segobin, Hélène Beaunieux, Anne‐Lise Pitel

**Affiliations:** ^1^ UNIROUEN, CRFDP Normandie Univ Rouen France; ^2^ UNICAEN, INSERM, PhIND, Physiopathology and Imaging of Neurological Disorders Institut Blood and Brain at Caen‐Normandie, Cyceron Caen France; ^3^ Louvain Experimental Psychopathology Research Group Psychological Science Research Institute, UClouvain Louvain‐la‐Neuve Belgium; ^4^ LPCN UR 7452 Université de Caen Normandie Caen France; ^5^ Service d'Addictologie Centre Hospitalier Universitaire de Caen Caen France; ^6^ Normandie Univ, UNICAEN PSL Université, EPHE, INSERM, U1077, CHU de Caen, GIP Cyceron, NIMH Caen France; ^7^ Institut Universitaire de France Paris France

**Keywords:** alcohol use disorder, executive functions, Korsakoff's syndrome, social cognition, theory of mind

## Abstract

**Background:**

Preliminary studies reported Theory of Mind (ToM) deficits in patients with Korsakoff's syndrome (KS). However, they presented several limits as they did not (1) control for key biasing factors (e.g., understanding of the task, amnesia); (2) compare KS with severe alcohol use disorder (sAUD) regarding ToM deficits; (3) explore the links between ToM abilities and other cognitive abilities. We thus directly compared cognitive ToM in patients with KS and sAUD, while considering task understanding and other cognitive deficits.

**Methods:**

Sixteen patients with KS, 70 patients with sAUD, and 69 healthy controls (HC) underwent a neuropsychological examination including a global cognitive screening, working memory and executive tests, as well as a cognitive ToM task designed to reduce cognitive load through the use of nonverbal materials (comic‐stories). The ToM task measured the ability to attribute first‐ and second‐order mental states to others and the level of understanding of the story with a control task.

**Results:**

We found no group effect on performance for the control condition. For both the first‐ and second‐order items of the ToM condition, HC performed significantly better than patients with sAUD and KS, who did not differ from each other. Results remained unchanged when controlling for the performance on the control task. However, when controlling for global cognitive status, patients with KS did not differ from HC anymore, contrary to patients with sAUD who remained altered. When controlling for executive/working memory performance, the main group effect was no longer observed. Flexibility was the only predictor of ToM performance in patients with sAUD.

**Conclusions:**

Cognitive ToM is similarly affected in patients with KS and sAUD, but global cognitive deterioration may underlie ToM deficits in patients with KS, whereas they may be more specifically related to flexibility impairments in patients with sAUD.

## Graphical abstract

The aim of this study was to explore Theory of Mind (ToM) in Korsakoff's syndrome (KS) compared with severe alcohol use disorder (sAUD). We used a cognitive ToM test that reduces the cognitive load and global cognitive screening, working memory, and executive tests. Results indicated that cognitive ToM deficits were similarly affected in KS and sAUD. However, they were related to global cognitive deterioration in patients with KS, but to flexibility impairments in patients with sAUD.

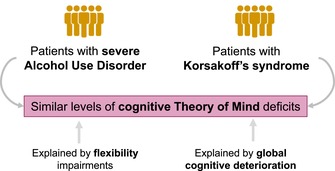

## INTRODUCTION

Korsakoff's syndrome (KS) is a persistent and debilitating neuropsychiatric disorder caused by thiamine deficiency and most frequently observed among patients with severe alcohol use disorder (sAUD), who have low thiamine intake, altered gastrointestinal thiamine absorption, and impaired thiamine metabolism (Arts et al., 2017). KS is characterized by severe episodic memory deficits, including anterograde amnesia and variable retrograde amnesia. Confabulations and false‐recognitions are also reported at the early stage of the disease. Episodic memory is affected out of all proportion to other cognitive functions, even though executive deficits can also be observed, potentially as a result of chronic and excessive alcohol consumption (Kopelman et al., 2009). In addition to the aforementioned cognitive deficits, patients with KS frequently exhibit altered abilities in emotional processing (Drost et al., 2019; Oscar‐Berman, 2012) and neuropsychiatric symptoms such as social, cognitive/behavioral, and emotional apathy (Oey et al., 2021; van Dorst et al., 2021), associated with social isolation and emotional loneliness (Oudman et al., 2018). Their socially maladaptive functioning, which includes difficulties understanding and engaging with others efficiently (Egger et al., 2002), may result from social cognition deficits.

Social cognition is indeed of paramount importance for the maintenance of high‐quality interpersonal relationships. Social cognition is defined as the ability to perceive, analyze, and respond to social situations (Brothers, 1990). The contemporary conceptual framework identified several components as emotion processing, theory of mind (ToM), attributional bias, and social perception and knowledge (Green et al., 2008). While self‐reported empathy appears preserved when experimentally tested (Oudman et al., 2021), the early clinical description of KS already mentioned a lack of interest toward other individuals' thoughts or emotions (Oscar‐Berman, 2012). The investigation of objective moral decision making (Oudman et al., 2021; Vlot et al., 2023) revealed a lower level of maturity and development in moral reasoning dilemmas in KS. ToM, which corresponds to the ability to infer cognitive or affective mental states to others, has also been very rarely studied in KS. Oosterman et al. (2011) examined the ability to make inferences and to take perspective on a story understanding task in 23 patients with KS compared to 15 healthy controls. Patients with KS presented lower results than controls regarding story understanding, despite good performance (>85% of correct answers). Moreover, the effect of perspective taking was similar in both groups, while the effect of story complexity was higher in patients with KS and mediated by executive dysfunction. More recently, Drost et al. (2019) evaluated both cognitive and affective components of ToM, using the Sally–Ann test (Baron‐Cohen et al., 1985) and the Faux Pas test of the mini‐SEA (Quesque et al., 2020) respectively, in 21 patients with KS and 21 healthy controls. For both tasks, patients with KS were described as being able to understand the storyline of the tasks (despite the authors reporting no comparative data on control/neutral questions added to check understanding of the story). Patients with KS performed lower than controls on both tasks, with a large effect size. They had thus impaired abilities to infer cognitive mental states to others and to judge whether a situation was embarrassing. In this study, executive deficits did not explain cognitive and affective ToM impairments in KS.

While these two investigations provided first evidence supporting the presence of ToM deficits in KS, they also have several limitations that hamper any conclusion on the specificity of ToM deficits in KS. First, both studies compared ToM abilities in KS using healthy participants as controls. Even though it is difficult in KS patients to dissociate deficits related to AUD or more inherently to KS pathology, in the absence of direct comparison between ToM performance in patients with KS and sAUD, it is even more complicated to consider whether ToM deficits are specific to KS, or whether they could result from excessive alcohol consumption and thus be shared with patients with sAUD. In effect, three systematic reviews and meta‐analyses showed ToM impairments in patients with sAUD compared to controls, with medium‐to‐large effect sizes (Bora & Zorlu, 2017; Hanegraaf et al., 2021; Onuoha et al., 2016). Second, these studies did not control for the level of understanding of the task and severe episodic memory deficits, which could lead to apparently altered ToM performance despite the absence of a genuine impairment of ToM processes. Third, the discrepancy observed in the literature regarding the relationships between executive functions and ToM abilities in KS may relate to the neuropsychological tasks chosen, namely disparate executive measures (response generation, planning, rule detection, and response inhibition) combined in a compound executive score and associated with a working memory measure (Oosterman et al., 2011) and a short screening test (the Frontal Assessment Battery, FAB; Drost et al., 2019). Further studies assessing each executive function separately are thus required.

To overcome these three limits, we examined whether (1) ToM processes are specifically impaired in patients with KS compared to sAUD, and (2) ToM processes are genuinely affected or are secondary by‐products of more general cognitive impairments (i.e., difficulty to understand the task, memory/executive dysfunctions). We used the false‐beliefs task (Desgranges et al., 2012; Duval et al., 2012; Fliss et al., 2016) and thus focused on cognitive ToM for three reasons. First, it has been designed and validated to reduce the cognitive load, notably on memory, language and executive functions, and had been used in severe episodic memory (Alzheimer's disease; Laisney et al., 2013 and semantic dementia; Duval et al., 2012). Second, it includes control questions to determine whether the assessment of ToM abilities is affected by story understanding. Third, it provides a comprehensive assessment of cognitive ToM with an evaluation of both first‐order (“I believe that X believes”) and second‐order (“I believe that X believes that Y believes”) components.

## MATERIALS AND METHODS

### Participants

We included 16 patients with KS, 70 patients with sAUD, and 69 healthy controls (HC). We matched groups on gender, but not on age and education. HC were younger than patients with sAUD (*p* = 0.01), who were younger than patients with KS (*p* < 0.001). HC had significantly more years of education than patients with sAUD (*p* = 0.004) and KS (*p* = 0.004), which did not differ.

Patients with KS fulfilled the DSM‐5 criteria for “alcohol‐induced major neurocognitive disorder, amnestic‐confabulatory type, persistent.” They were recruited as inpatients at Caen University Hospital (*N* = 9) and in a nursing home dedicated to patients with KS (Maison Vauban, Roubaix, France, *N* = 7). We included each patient after a careful selection procedure involving the confirmation of severe episodic memory deficits on the French version of the Free and Cued Selective Reminding Test (Van der Linden et al., 2004) (Table 2) and an individual examination by experts in neuropsychology and behavioral neurology to ensure that they met the inclusion criteria. In addition, clinical and neuroimaging investigations ruled out other possible causes of memory impairment (particularly focal brain damage or neurodegenerative disease, Maillard et al., 2021). All patients with KS had a history of heavy drinking, but their amnesia made it difficult to obtain accurate information about their past alcohol consumption. The background information collected for the patients with KS came mainly from family members and medical records.

Clinicians recruited patients with sAUD among inpatients in the Addiction Department at Caen University Hospital. Patients met “alcohol dependence” criteria according to the DSM‐IV‐TR (American Psychiatric Association (APA), 2000) or “severe AUD” criteria according to the DSM‐5 (i.e., the presence of six or more diagnosis criteria; American Psychiatric Association, 2013). None of them presented physical symptoms of alcohol withdrawal as assessed by the Cushman's scale (Cushman et al., 1985) at inclusion. We included patients with sAUD at least 48 h after the last diazepam prescription (according to the half‐life of the benzodiazepine used). They were interviewed using a modified version of the semi‐structured lifetime drinking history and the Alcohol Use Disorders Identification Test (AUDIT; Gache et al., 2005). Table 1 presents alcohol‐related variables.

**TABLE 1:** Description of the participants.

	HC (*N* = 69)	Patients with sAUD (*N* = 70)	Patients with KS (*N* = 16)	Statistics	Post hoc comparisons
**Demographical variables**					
Age	42.4 ± 8.35 [25–65]	47 ± 8.3 [33–67]	56.4 ± 5 [49–67]	*F*(2,152) = 16.6 *p* < 0.001	HC < sAUD < KS
Gender (% male)	59.42%	70%	43.75%	χ^2^ = 4.36 *p* = 0.1	HC = sAUD = KS
Education	12.6 ± 2.7 [8–20]	11.30 ± 1.7 [8–15]	10.4 ± 2.2 [8–15]	*F*(2,152) = 9.51 *p* < 0.001	HC > (sAUD = KS)
**Psychological variables**					
STAI‐A	26.6 ± 6.6 [20–47]	31.90 ± 10.7 [20–61]	35.50 ± 14.6 [20–66]	*F*(2,146) = 7.73 *p* < 0.001	HC < (sAUD = KS)
STAI‐B	32.6 ± 7.3 [20–50]	44.40 ± 11.50 [24–72]	37.90 ± 10.3 [24–57]	*F*(2,146) = 25.4 *p* < 0.001	HC < sAUD HC = KS sAUD = KS
BDI	3.7 ± 4 [0–21]	12.4 ± 7.2 [0–28]	7 ± 6.1 [0–19]	*F*(2,149) = 37.3 *p* < 0.001	HC < sAUD HC = KS KS < sAUD
**Substance‐related variables**					
AUDIT	3.3 ± 2.8 [0–16]	29.4 ± 6.5 [9–40]	NA	*t* = 30.2 *p* < 0.001	HC < sAUD
Duration of alcohol use disorder (years)	/	10.4 ± 7.9 [0–34]	NA	/	/
Number of previous alcohol withdrawal episodes	/	2.8 ± 1.9 [0–11]	NA	/	/
Abstinence (number of days before inclusion)	/	12.9 ± 6.7 [4–50]	NA	/	/
Daily alcohol consumption before treatment (in standard drinks^a^)	/	17.8 ± 11.6 [0–80]	NA	/	/
Fagerström^b^	1.22 ± 2.26 [0–8]	4.41 ± 3.27 [0–14]	2.43 ± 2.79 [0–10]	*F*(2,128) = 17.7 *p* < 0.001	HC < sAUD HC = KS KS = sAUD

*Note*: The mean ± standard deviation are reported.

Abbreviations: /, not applicable; AUDIT, Alcohol Use Disorder Identification Test; BDI, Beck Depression Inventory; HC, healthy controls; KS, patients with Korsakoff's Syndrome; NA, data not available; sAUD, patients with severe alcohol use disorder; STAI, State–Trait Anxiety Inventory.

^a^ A standard drink corresponds to 10 g of pure ethanol in France. For this variable, there were missing data for 11 patients with sAUD because they were not able to report this information due to the severity of cognitive deficits.

^b^ For this variable, data were collected for 68 patients with sAUD, 14 patients with KS, and 50 HC.

HC presented low‐risk alcohol consumption (AUDIT score < 7 for men and <6 for women) and did not present severe depressive (Beck Depression Inventory score < 29, Beck et al., 1961) nor dementia symptoms (Mattis Dementia Rating score < 129, Mattis, 1976).

Participants did not present previous neurological, psychiatric, or history of brain injury (except brain abnormalities associated with sAUD and KS) or other substance use disorders (except tobacco for all participants). Patients with sAUD and HC were free from any psychotropic medication, while four patients with KS remained under long‐term psychotropic medication (Laniepce et al., 2023). A detailed description of medications in the four patients with KS included in the nursing home is available in the Table S1. All participants completed questionnaires related to state (STAI‐A) and trait (STAI‐B) anxiety (Spielberger et al., 1983), as well as depression (BDI). Table 1 presents psychological variables.

All participants underwent a neuropsychological examination. We used the Mini Mental State Examination (MMSE; Folstein et al., 1975) to examine global cognitive status. We also measured: (1) Inhibition using the time in seconds needed to complete the interference condition minus the time needed for the denomination condition of the Stroop task (Stroop, 1935); (2) Flexibility using the time taken to complete part B of the Trail Making Test minus the time taken to complete part A (Reitan, 1955); and (3) Working memory using the raw score of the verbal backward span of the WAIS‐III (Wechsler, 1997). Results showed global cognitive deterioration, as well as flexibility and inhibition deficits in patients with KS, and a working memory impairment in patients with sAUD (see Table 2 for detailed information).

**TABLE 2:** Neuropsychological performance in HC, patients with sAUD, and patients with KS.

Cognitive function	Task	HC (*N* = 69)	Patients with sAUD (*N* = 70)	Patients with KS (*N* = 16)	Statistics	Post hoc comparisons
Global cognitive status	MMSE (total score)	28.5 ± 1.5 [24–30]	27.6 ± 2.1 [20–30]	23.3 ± 2.7 [18–27]	*F*(2,149) = 33.84 *p* < 0.001	(HC = sAUD) > KS
Episodic memory	Delayed free recall (FCSRT)	13.1 ± 2.2 [6–16]	11 ± 3 [0–16]	2.5 ± 3 [0–11]	*F*(2,150) = 69.22 *p* < 0.001	HC > sAUD > KS
Inhibition	Stroop task (s)	47.8 ± 19.8 [13–143]	67.5 ± 44.9 [12–308]	103 ± 62.5 [39–262]	*F*(2,146) = 6.17 *p* = 0.003	HC = sAUD HC < KS sAUD = KS
Flexibility	Trail Making Test (s)	40.4 ± 23.8 [7–130]	82.9 ± 112 [19–890]	135 ± 90.2 [25–296]	*F*(2,146) = 4.13 *p* = 0.01	
Working memory	Verbal backward span (raw score)	5.21 ± 1.4 [3–8]	4.23 ± 1.3 [2–7]	4.06 ± 1 [3–7]	*F*(2,149) = 6.52 *p* = 0.002	HC > sAUD HC = KS sAUD = KS

*Note*: Group effects were tested with ANCOVAs with age and education as covariates followed by post hoc comparisons using Bonferroni post hoc tests. The mean ± standard deviation are reported.

Abbreviations: FCSRT, Free and Cued Selective Reminding Test; MMSE, Mini Mental State Examination.

All participants were informed about the study approved by the local ethics committee of Caen University Hospital (CPP Nord Ouest III, no. IDRCB: 2011‐A00495‐36) prior to their inclusion and gave their written informed consent. For patients with KS, we obtained informed consent from caretakers or caregivers as well as from patients themselves.

### Methods

#### The false‐beliefs task

**FIGURE 1:**
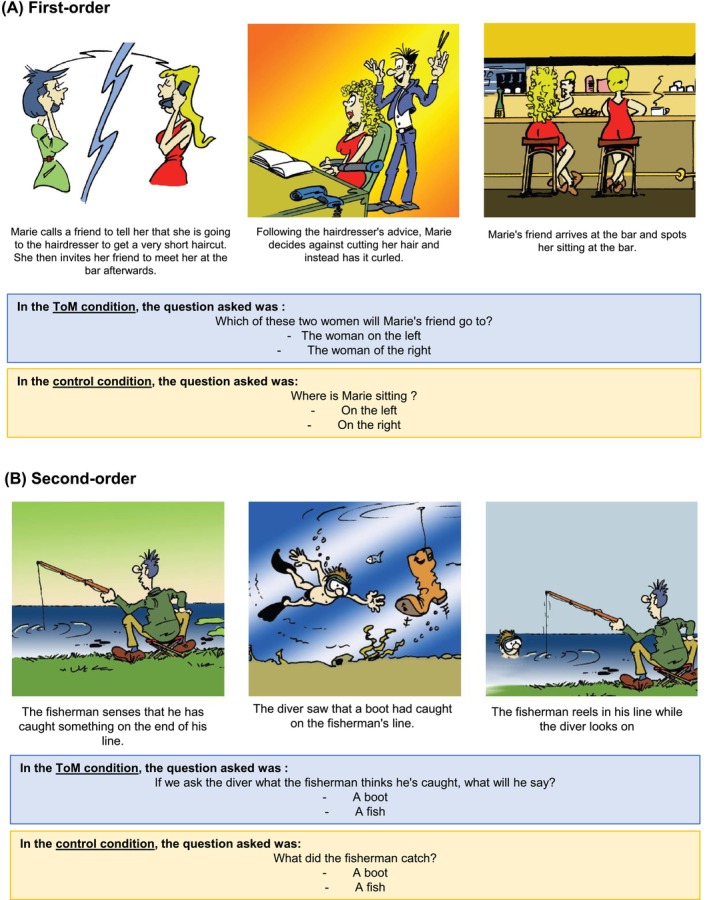
Example of story used in the false‐belief task, adapted from Desgranges et al. (2012). (A) A first orer item. (B) Second orer item. ToM, theory of mind.

We used the false‐beliefs task, a revised version from the Wimmer and Perner's false‐belief task (Wimmer & Perner, 1983), to assess cognitive subcomponents of ToM, specifically the ability to attribute first‐ and second‐order mental states to others (Desgranges et al., 2012; Duval et al., 2012; Fliss et al., 2016). This task consists of the presentation of 15 short comic strips representing everyday situations in which a character is involved, leading one of the characters to form an erroneous belief about the actual state of the world. In order to reduce the cognitive load and the involvement of other cognitive functions likely to interfere with theory‐of‐mind abilities, each story is divided into three parts, presented on the same page in the form of three‐color drawings, each accompanied by a verbal caption (Figure 1). The first part of the story describes a material or interpersonal situation in which a character discovers something. In the second part, the situation changes without the character's knowledge. The third part then presents the situation in which the character's belief is incorrect. After looking at the drawings and reading the captions, participants must answer the question at the bottom of the page, by selecting the correct answer from two options (Desgranges et al., 2012). This task consisted of two conditions. First, in the ToM condition, participants were asked about the beliefs of one of the characters in the story. Eight of the 15 comics contained first‐order representations (“X thinks that…”; Figure 1A) and seven second‐order representations (“X thinks that Y thinks that …”; Figure 1B). Second, in the control condition, the same comic strips were used, but the question probed participants' understanding of the cartoon scenario without involving any ToM ability. In both conditions, the participant was asked to answer the question written on the board, by choosing between two suggestions. Participants did not receive any feedback. Performance was expressed as the number of correct answers for each condition: 1st order (/7), 2nd order (/8) and control condition (/15).

### Statistical analyses

We performed all statistical analyses using Jamovi 2.2.21 (The Jamovi Project, 2025) with a *p* value set at 0.05.

First, we examined the group effect (HC vs. sAUD vs. KS) on the control condition of the false‐beliefs task using an ANCOVA with age and education as covariates.

Second, we conducted a mixed‐effects model to examine the effects of group and order (1st vs. 2nd order) on performance on the ToM condition, with age and education as covariables. The model included group and order as fixed effects, and participants for the random intercepts. When significant main effects emerged, we performed Bonferroni post hoc tests.

Third, we examined whether ToM abilities observed in patients were genuinely affected or secondary to other cognitive dysfunction. To this end, we performed other mixed‐effects models by including separately additional covariates: understanding of the task, global cognitive status and executive/working memory.

## RESULTS

### Between‐group comparisons on the false‐beliefs task

Detailed scores on the false‐beliefs task are reported in Table 3. When controlling for age and education, the ANCOVA did not reveal any significant group effect on performance for the control condition [*F*(2, 150) = 1.76, *p* = 0.17].

**TABLE 3:** Performance on the false‐beliefs task in HC, patients with sAUD, and KS.

	HC (*N* = 69)	Patients with sAUD (*N* = 70)	Patients with KS (*N* = 16)	Post hoc comparisons
Control task (/15)	14.4 ± 1.3 [7–15]	13.7 ± 1.8 [6–15]	12.9 ± 1.81 [8–15]	HC = sAUD = KS
First order (/7)	7.3 ± 1 [2–8]	6.5 ± 1.4 [2–8]	5.7 ± 2.2 [1–8]	HC > (sAUD = KS)
Second order (/8)	5.9 ± 1.1 [2–7]	4.7 ± 1.6 [0–7]	4.4 ± 1.9 [1–7]	HC > (sAUD = KS)
Total score (/15)	13.2 ± 1.9 [5–15]	11.3 ± 2.4 [2–15]	10.1 ± 3.7 [3–15]	HC > (sAUD = KS)

*Note*: The mean ± standard deviation are reported. Post hoc analyses concern the group effect (HC vs. sAUD vs. KS) with age and education as covariates.

For the ToM condition, when controlling for age and education, the mixed‐effects model revealed a significant group effect on performance (*F*(2, 151) = 8.64, *p* < 0.001). Bonferroni post hoc analyses showed that HC performed significantly better than patients with sAUD (*p* < 0.001) and KS (*p* = 0.008), who did not significantly differ from each other (*p* = 1). The effect of order was also significant (*F*(1, 152) = 104.74, *p* < 0.001) with significantly better performance on the first‐order than on the second‐order items. However, the interaction between group and order effects was not significant (*F*(2, 152) = 1.02, *p* = 0.36), indicating that the second‐order condition was not disproportionately affected in the patient groups.

### Genuine or secondary ToM deficits

#### Understanding of the task

When additionally controlling for the understanding of the task, the group effect remained significant (*F*(2, 150) = 6.70, *p* = 0.002) with similar results on the post hoc comparisons (HC > sAUD (*p* = 0.003), HC > KS (*p* = 0.02), sAUD = KS (*p* = 1)).

#### Global cognitive status

When additionally controlling for global cognitive status, the group effect remained significant (*F*(2, 148) = 4.58, *p* = 0.01). However, Bonferroni post hoc analyses showed that HC still performed significantly better than patients with sAUD (*p* = 0.01), but no difference was observed between HC and patients with KS (*p* = 1), who did not significantly differ from patients with sAUD (*p* = 0.81).

#### Executive and working memory abilities

When additionally controlling for executive and working memory abilities, the group effect was not significant anymore (*F*(2, 135) = 2.59, *p* = 0.07) even though there is a tendency. With an exploratory approach, we conducted a stepwise linear regression to examine the extent to which *flexibility, inhibition*, and *working memory* predicted performance on the cognitive *ToM performance in patients with sAUD*. The overall model was statistically significant (*F*(3, 62) = 4.36, *p* < 0.008), and explained approximately 17.4% of the variance in the ToM performance (*R* ^*2*^ = 0.134). Among the predictors, *flexibility* emerged as a significant negative predictor (*β* = −0.34, *p* = 0.009), indicating that higher time to perform TMT‐B minus TMT‐A task, reflecting flexible difficulty, was associated with lower total scores. *Inhibition and working memory* did not significantly contribute to the model (*β* = 0.09, *p* = 0.45 and *β* = 0.19, *p* = 0.11 respectively).

## DISCUSSION

Our study confirms the presence of cognitive ToM deficits in patients with KS (Drost et al., 2019; Oosterman et al., 2011) and deepens our understanding of this social cognition impairment in this severe and debilitating disease. First, ToM is quantitatively affected to the same extent in patients with KS and sAUD, whatever the complexity level of the cognitive ToM concerned, even when controlling for the level of understanding of the story and when avoiding any involvement of episode memory. Second, the cognitive processes underlying ToM deficits in KS and sAUD seem to be qualitatively different. Beyond the similarity between KS and sAUD at the quantitative level, our data indeed suggest that the cognitive processes underlying ToM deficits differ in the two clinical forms: It appears to be secondary to global cognitive deterioration in patients with KS, whereas it seems consecutive to executive and working memory performance in patients with sAUD.

The two groups were similarly impaired when compared to HC and were affected identically for first‐ and second‐order conditions, thus independently of the complexity of the ToM task. While the presence of ToM deficits in sAUD is now well‐described (Bora & Zorlu, 2017; Hanegraaf et al., 2021; Onuoha et al., 2016), the present study is the first to directly compare ToM performance in patients with KS and sAUD. Our findings show that ToM abilities assessed by a false‐beliefs task do not allow differentiating patients with KS from patients with sAUD, and therefore do not seem to serve as a reliable marker of KS.

Contrary to the two previous investigations of ToM in KS (Drost et al., 2019; Oosterman et al., 2011), we found that cognitive ToM performance cannot be explained by understanding difficulties, as groups did not significantly differ on the control condition. In the same vein, we intentionally chose a cognitive ToM task designed to be used in patients with severe episodic memory deficits since it compensates for amnesia by using comics (Desgranges et al., 2012). Even in that context, KS patients had impaired ToM results, indicating that cognitive ToM deficits should not be regarded as a mere consequence of amnesia. However, when we controlled for the global cognitive deterioration, we showed that patients with KS did not differ from controls anymore, unlike patients with sAUD who remain impaired. These findings are in favor of a processual specificity of ToM impairments in KS compared to sAUD and suggest that the cognitive processes underlying ToM deficits are different in patients with KS and sAUD. In that theoretical context, in patients with KS, cognitive ToM deficits reflect more general and cognitively unspecific relationships with different cognitive measures including executive and working memory, as indicated by the analysis including the global cognitive status. Taken together, ToM deficits in patients with KS appear thus to be secondary, explained by the global cognitive impairment, encompassing executive and WM deficits, for example.

In patients with sAUD, executive dysfunction and working memory deficits are commonly reported (Oscar‐Berman, 2012; Schmid et al., 2021). When controlling for these cognitive processes, our results indicated that the patients did not differ from controls on cognitive ToM abilities anymore. This suggests that the cognitive ToM deficit in patients with sAUD is influenced by executive functions, supporting prior findings (Maurage et al., 2015; Schmid et al., 2022; Thoma et al., 2013). Relationships between executive functions and inference abilities have previously been described in both KS (Oosterman et al., 2011) and sAUD (Schmid et al., 2021), whereas the contribution of working memory performance has rarely been investigated or has not been fully exploited (Oosterman et al., 2011). Our data are consistent with Samson's cognitive model of ToM (Samson, 2009), which emphasizes the central role of working memory, inhibition, and flexibility in inferring the mental states of others. More specifically, our study indicated relationships between flexibility and ToM performance in patients with sAUD. This result is in agreement with an earlier investigation that identified a relationship between flexibility and faux‐pas results in patients with sAUD (Schmid et al., 2022). Flexibility may be needed to simultaneously consider the actions and mental states of two different story characters, which is crucial to answer correctly to the false‐belief task (Schmid et al., 2022). However, these results should be interpreted with caution since a tendency remained regarding the group effect when controlling for executive and working memory results, and flexibility explained only a small part of the ToM deficits. Executive dysfunction and working memory impairments may thus either partially account for or exacerbate the ToM deficit. This highlights a complex interplay between executive functions and ToM, warranting further exploration using a variety of tasks targeting both domains.

As patients with sAUD were included early in abstinence, this may result from the harmful effects of chronic and excessive alcohol consumption and/or alcohol withdrawal. A second hypothesis is that these cognitive ToM deficits may be present before the development of AUD, possibly as a vulnerability factor. They would thus resist to global cognitive recovery as showed in a longitudinal investigation (Rupp et al., 2012). In the present study, our cross‐sectional design limits our interpretation and further longitudinal studies are required.

The present study has several strengths, including a detailed neuropsychological assessment in a large number of patients with sAUD and KS, and an assessment of cognitive ToM using the false‐beliefs task, which allows us to control for the level of understanding and for severe memory deficits. While our method is particularly relevant for investigating cognitive ToM in KS without being biased by amnesia, our results should be interpreted with caution when applying them in clinical settings. In our everyday environment, situations involving social cognition may be modulated by episodic memory abilities (Moreau et al., 2013; Spreng, 2013). For example, if you experience that a person is sensitive and has been involuntarily offended by your behavior, you will use this memory to adjust your social cognition and thus your behavior for the upcoming interactions. Contrary to patients with sAUD, patients with KS who have amnesia will not be able to use prior experience to modulate or adjust their social cognition behavior, potentially resulting in a more severe alteration of social behavior in everyday life. These relationships are of high importance for social support, which is particularly crucial for individuals who are dealing with the challenges of alcohol consumption and cognitive impairments. Fidder et al. (2023) emphasized that KS patients show significant difficulties in interpreting social cues and understanding others' perspectives, which further complicates their social interactions and may contribute to social isolation. Appropriate social interactions can help to mitigate the impact of cognitive deficits and reduce relapse risk. Conversely, impairments in social cognition can result in social isolation, interpersonal conflicts, and increased stress, all of which can exacerbate both the cognitive and alcohol‐related problems experienced by KS patients (Montagne et al., 2006).

This study has several limitations. First, when making a direct comparison between patients with sAUD and KS, it is important to keep in mind that patients with KS inherently include impairments associated with both AUD and KS‐related pathology. As a result, it may be difficult to clearly disentangle the individual contributions of each condition to the observed results. Additionally, the KS sample size was relatively small (*N* = 16 patients), reflecting the strict diagnostic criteria used to ensure that participants had persistent anterograde amnesia. Furthermore, the KS sample included a higher proportion of women due to the recruitment notably conducted in a KS nursing home dedicated to women. It was also, on average, older than the other groups, which explains why age was systematically used as a covariate. Our study provides novel findings on cognitive ToM, but further research is needed to examine affective ToM. Finally, with regard to the relationship between cognitive functioning and ToM performance, the absence of variability for episodic memory results hampered the exploration of correlations. In terms of global cognitive status, we used the MMSE, a broad screening tool known to be influenced by various cognitive abilities such as verbal abilities, executive functions, or working memory. Consequently, our findings in KS may reflect general cognitive impairment or be driven by more specific deficits not specifically evaluated in the present study.

Taken together, our findings indicate that despite the similar severity of cognitive ToM deficits in patients with KS and sAUD, the underlying cognitive mechanisms vary in the two clinical forms. While it seems to be secondary to the overall cognitive deterioration in patients with KS, it may rather be explained by executive and working memory performance in patients with sAUD, notably mental flexibility. For clinicians, these findings suggest that it is relevant not to limit oneself to a quantitative approach to ToM deficits, but also to adopt a qualitative approach by exploring the cognitive nature underlying the deficit.

## FUNDING INFORMATION

This work was supported by the French National Institute for Health and Medical Research (INSERM), the French National Agency for Research (ANR) and Conseil Regional de Normandie.

## CONFLICT OF INTEREST STATEMENT

The authors report no competing interests.

## Supporting information


Table S1

## Data Availability

The data that support the findings of this study are available from the corresponding author upon reasonable request.
