# Supplementary material for: Theory of mind deficits in Korsakoff's syndrome and alcohol use disorder: Similar deficits but different underlying cognitive processes
Source: Alcohol Clin Exp Res (Hoboken). 2025 Aug 22;49(9):1962–71. doi: 10.1111/acer.70135 (PMC12463770; doi:10.1111/acer.70135)
Supplement: Supplementary file 1 — Table S1 [file ACER-49-1962-s001.docx]

**Supplementary Table 1 : Individual prescription in patients with KS.**

| KS | Gender, Age | Major medication (Daily dose) |
| --- | --- | --- |
| 2 | W, 51 | Zopiclone (7.5 mg/day), Alprazolam (1.5 mg/day) |
| 3 | W, 52 | Hydroxyzine (150 mg/day), Lorazepam (3 mg/day),  Duloxetine (60 mg/day) |
| 4 | W, 53 | Clonazepam (0.9 mg/day) |
| 5 | W, 61 | Venlafaxine (150 mg/day), Lorazepam (1 mg/day) |

*Among the 16 patients with KS included in the present study, 4 were taking medication. These data are also available in Laniepce et al., 2023.*
